# Unusual Malposition of a Chest Tube, Intrathoracic but Extrapleural

**DOI:** 10.1155/2018/8129341

**Published:** 2018-08-06

**Authors:** Alqasem Fuad H. Al Mosa, Mohammed Ishaq, Mohamed Hussein Mohamed Ahmed

**Affiliations:** ^1^ATA, King Faisal Specialist Hospital & Research Centre (KFSH&RC), Riyadh, Saudi Arabia; ^2^Surgery, King Faisal Specialist Hospital & Research Centre (KFSH&RC), Riyadh, Saudi Arabia; ^3^Organ Transplant Centre, King Faisal Specialist Hospital & Research Centre (KFSH&RC), Riyadh, Saudi Arabia

## Abstract

Chest tube malpositioning is reported to be the most common complication associated with tube thoracostomy. Intraparenchymal and intrafissural malpositions are the most commonly reported tube sites. We present a case about a 21-year-old patient with cystic fibrosis who was admitted due to bronchiectasis exacerbation and developed a right-sided pneumothorax for which a chest tube was inserted. Partial initial improvement in the pneumothorax was noted on the chest radiograph, after which the chest tube stopped functioning and the pneumothorax remained for 19 days. Chest computed tomography was done and revealed a malpositioned chest tube in the right side located inside the thoracic cavity but outside the pleural cavity (intrathoracic, extrapleural). The removed chest tube was patent with no obstructing materials in its lumen. A new thoracostomy tube was inserted and complete resolution of the pneumothorax followed.

## 1. Introduction

Tube thoracostomy has become a common surgical procedure with relatively safe outcomes [1]. The complications include malposition, infection, and organ injury with chest tube malposition being the most common complication [2]. Not all malpositioned chest tubes are clinically significant. Different studies described the locations of malpositioned chest tubes by means of CT imaging. In one study looking at complications after emergency tube thoracostomy utilizing CT scan, chest tube malposition was the most common complication, which was observed in 26% of the patients (20 out of 77 tubes). Eighteen intrathoracic (5 intraparenchymal, 9 intrafissural), and two extrathoracic malpositioned tubes were seen by CT imaging [3].

## 2. Case Presentation

The patient is a young male with cystic fibrosis, combined obstructive and restrictive lung disease with bilateral bronchiectasis with poor compliance to medications and chest physiotherapy.

The patient has a chronic respiratory illness with declining lung function. He is on CF treatment and was following in the pulmonology and lung transplant clinic for his disease.

**Figure 1:**
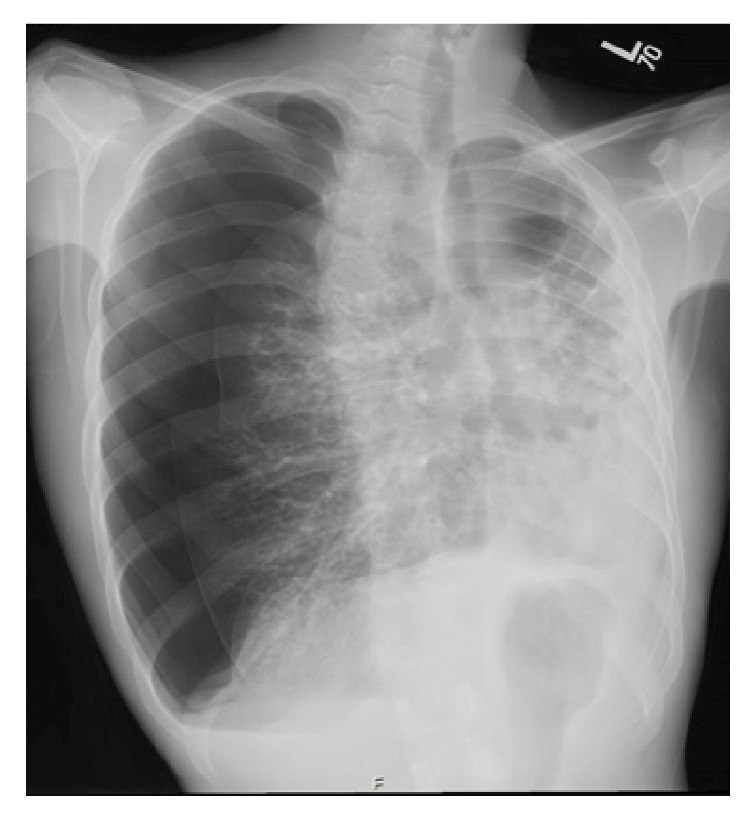
Chest X-ray on admission showing a right-sided pneumothorax prior to insertion of the chest tube.

On admission the patient had a right-sided pneumothorax on chest X-ray (Figure 1). He had chronic respiratory symptoms with cough, sputum, and shortness of breath that was associated with pleuritic chest pain. On examination, the patient was stable with oxygen saturation of 91% on room air. He had absent breath sounds on right side with normal cardiovascular and abdominal examination.

His laboratory work was unremarkable. His chest X-ray (Figure 1) showed significant right-sided pneumothorax with partial collapse of the right lung. Severe destruction of the lungs with bronchiectasis and atelectasis was noted. The mediastinum and heart were shifted to the left side.

The patient was admitted and was given broad-spectrum antibiotics based on previous culture results. A right-sided chest tube of size 24 Fr was inserted in the emergency room under aseptic measures.

**Figure 2:**
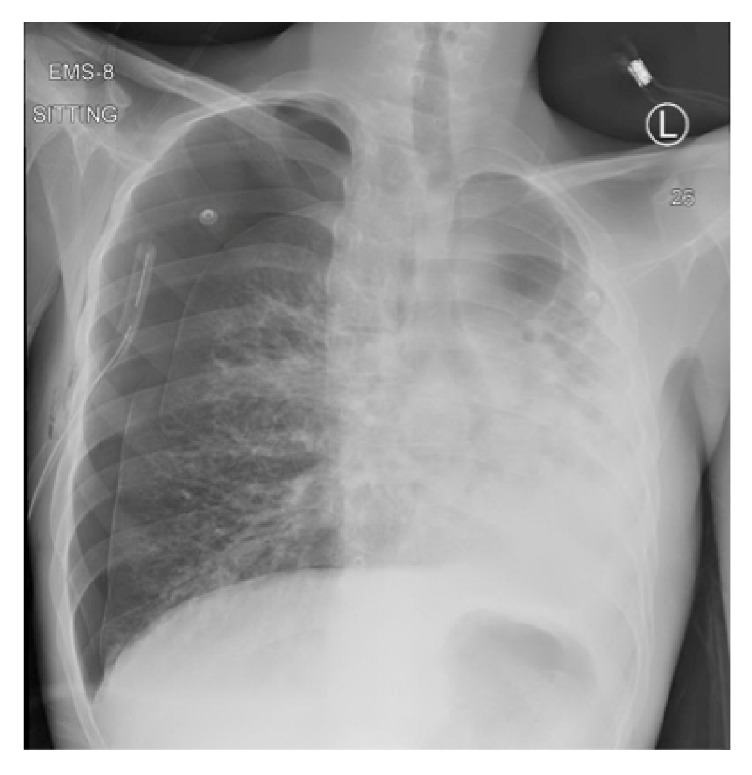
Chest X-ray after insertion of the chest tube showing improvement in the right-sided pneumothorax with subcutaneous emphysema.

The chest X-ray (Figure 2) after the chest tube insertion showed interval improvement of the pneumothorax with subcutaneous emphysema near the chest tube insertion site.

**Figure 3:**
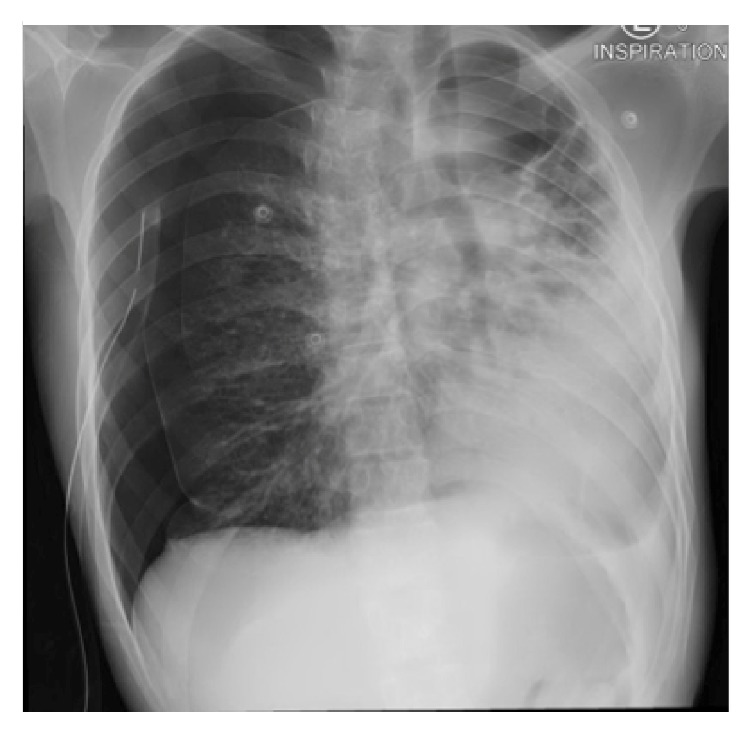
Chest X-ray showing worsening of the pneumothorax despite presence of a chest tube.

Repeated chest X-rays showed interval improvement of the pneumothorax, and the pneumothorax remained relatively unchanged for about a week, but then it worsened (Figure 3).

**Figure 4:**
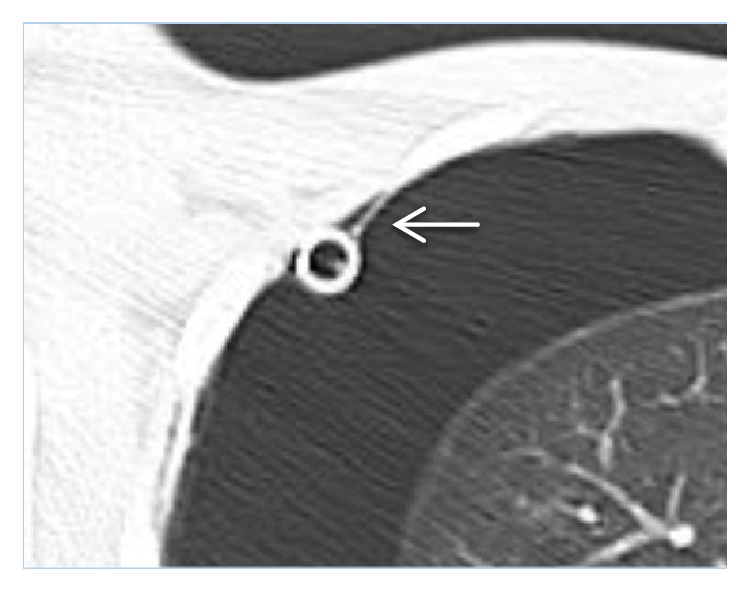
Chest CT axial cut magnified showing the location of the chest tube in relation to the chest wall and parietal pleura. White arrow: parietal pleural.

**Figure 5:**
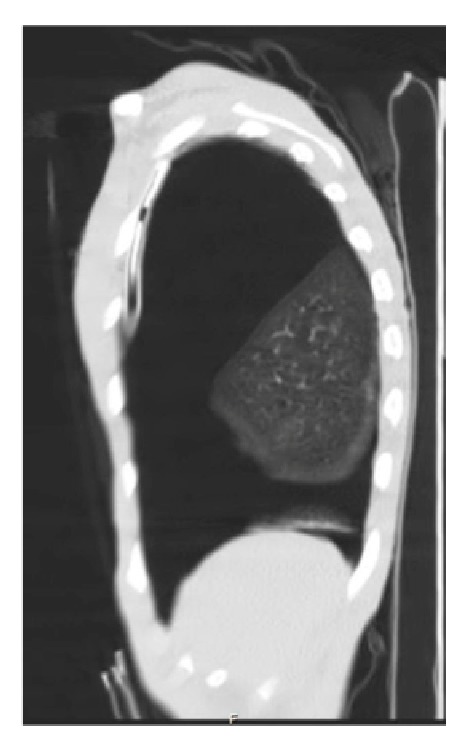
Chest CT scan sagittal cuts showing the chest tube location.

After worsening, the pneumothorax remained stable for 10 days. During that time, a chest CT was done which showed diffuse bronchiectasis in the right lung and bronchial wall thickening with partial atelectasis of the right lower lobe. The left lung was collapsed and showed diffuse bronchiectasis with cystic changes. The large right-sided pneumothorax was seen. The chest tube was noted to be intrathoracic but extrapleural with the parietal pleura shown as thin layer in some of the transverse cuts (Figure 4). Sagittal and coronal planes show the chest tube inside the chest cavity but in the extrapleural space adherent to the inner side of the right chest wall (Figure 5).

Manipulation of the chest tube (stripping, flushing, and aspirating) resulted in partial regain of tube function for 24 to 48 hours and observation of oscillation with breathing. The tube stopped functioning afterwards.

**Figure 6:**
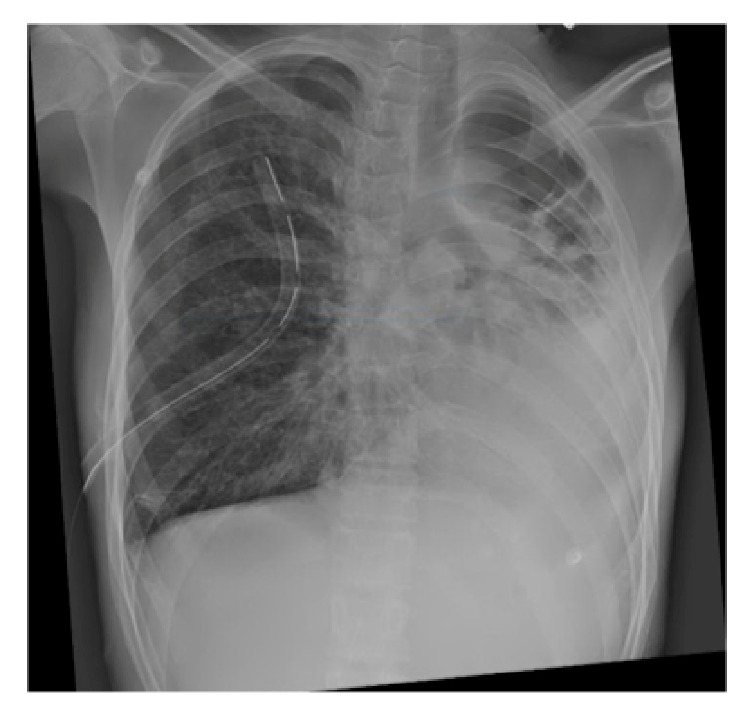
Chest X-ray after insertion of a new chest tube and resolution of the right-sided pneumothorax.

The chest tube remained for nineteen days before it was changed. The old tube was removed, and it was noted that it does not have any obstructing materials in its lumen. A new 28 Fr rigid chest tube was inserted under aseptic technique and connected to underwater seal with wall suction. A chest X-ray obtained after insertion revealed total resolution of the pneumothorax (Figure 6).

Four days later, the patient was discharged home after removal of the chest tube in a stable condition with follow-up in the clinic.

## 3. Discussion

Chest tube malposition is the most common complication related to chest tube insertion [2], with intraparenchymal and intrafissural locations being the most common. Different studies have demonstrated the ability of computed tomography imaging to describe the location of a malpositioned chest tube. We report on a rare location of a malpositioned chest tube that is intrathoracic but extrapleural.

Different studies described the locations of malpositioned chest tubes by means of CT imaging. In one study, CT imaging of emergency tube thoracostomy was positive for malpositioned chest tubes in 28 out of 76 chest tubes inserted. The locations described were as follows: 23 tubes in the intrathoracic location (20 intraparenchymal, 3 intrafissural) and 5 tubes in the extrathoracic location (4 in mediastinum, 1 in chest wall) [4].

In a CT pictorial review of tube thoracostomy, malpositioned chest tubes were found to be placed in intraparenchymal, intrafissural, mediastinal, and abdominal locations [5].

In a prospective study, chest tube malposition in critically ill patients was identified in 35 patients (30% of 106 chest tubes in total). Twenty-two chest tubes (21%) were diagnosed as being intrafissural, and ten (9%) were diagnosed as being intraparenchymal (9%). One tube was described as being intrathoracic but extrapleural which was inserted by a trocar and was replaced by another chest tube because it was not draining a pneumothorax [6].

In a 2013 paper enrolling 42 patients studying the results of emergency placement of chest tubes, malpositioning of chest tubes was detected by CT scan in 9 cases [7].

Multiple case reports have described rare malpositioning and complications of chest tube placement. An example of such complications is heart puncture [8, 9].

An example of a life-threatening intra-abdominal chest tube related malpositioning and complication is presented in a case report published in 2015 in which a life-threatening hemoperitoneum and liver injury occurred after chest tube insertion [10].

The authors of the paper were able to find one study describing a similar location [6] with brief description of the clinical presentation of the patient.

We believe that the initial improvement in the pneumothorax resulted from opening the pleural space during the insertion of the chest tube causing the release of an initial gush of air and hence initial temporary improvement of the pneumothorax; however, the chest tube was inserted subpleurally and not through the pleural opening. There could also have been some temporary communication between the drain and the pleural space which gradually closed off.

A couple of thoracic surgeons in our unit have noted possible similar previous observations of this malpositioning in a patient with thickened pleura. It is known that there are varying degrees of pleural involvement in cystic fibrosis patients due to chronic pleural inflammation secondary to chronic pulmonary infections. Pulmonary involvement in this patient with cystic fibrosis could explain the unusual malpositioning of the chest tube despite proper insertion technique.

## 4. Conclusion

We present a case about a malpositioned chest tube in a young male patient suffering from cystic fibrosis. The tube was intrathoracic but extrapleural in location. Patients with pleural pathology, like cystic fibrosis patients, could be prone to unusual malpositioning of a chest tube secondary to pleural inflammatory involvement in chronic pulmonary infections. Care and proper techniques are paramount to preventing such complications. It is important to determine why a chest tube is not functioning and to insert a new chest tube as opposed to keeping a nonfunctioning one in situ for prolonged periods. Keen and prompt review of CT chest scans can provide great insight on whether or not a chest tube is malpositioned.
